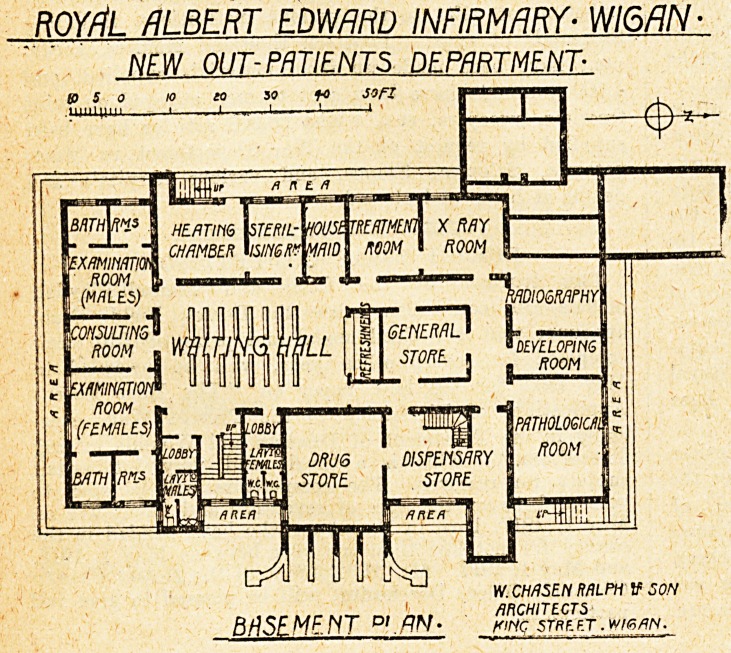# Royal Albert Edward Infirmary, Wigan

**Published:** 1916-12-30

**Authors:** 


### HOSPITAL ARCHITECTURE AND CONSTRUCTION.

### Royal Albert Edward Infirmary, Wigan.

### NEW OUT-PATIENTS' BLOCK.

THIS new building is a detached block connected with the main building by a corridor. There are two storeys, a basement and a ground floor, and as the ground floor is apparently some 4 to 5 feet above the pavement level, the basement storey is to some extent above ground. The entrance for patients is in the centre of the east front, and this entrance also forms the exit for patients after getting their medicines. Immediately inside is a lobby. Here incoming patients pass the porter's office and registry, and into the large waiting-hall. Arranged at the north and south ends are the medical and surgical consulting-rooms, with the usual examining and dark-rooms for the medical patients, and examining and dressing-rooms for the surgical patients. On the west side is a small theatre for minor operations, with preparation and recovery rooms. Adjoining is the nurses' room, with store and lavatory. A separate drive-in on the north gives access for patients to a receiving-room, close to which is the medical officer's room. Separate sanitary offices for each sex are placed on the east side. The dispensary is placed on the east side, and has a small waiting-space for patients.

GROUND FLOOR PLAN·

BASEMENT PLAN·

The basement has a large central waiting-hall, in the centre of which is a refreshment bar and a general store. At the south end is the consulting-room for the doctor in charge of diseases of the skin, with an examination-room for male and one for female patients, and two bathrooms for each sex. On the west side is the *x-ray* room, with room for electrical treatment, and on the north a radiography-room with dark room attached and a large pathological-room. The remainder of the basement consists of stores for drugs, heating-chamber, and sterilising-room, etc.

The architects were Messrs. W. C. Ralph and Son.

## Figures and Tables

**Figure f1:**
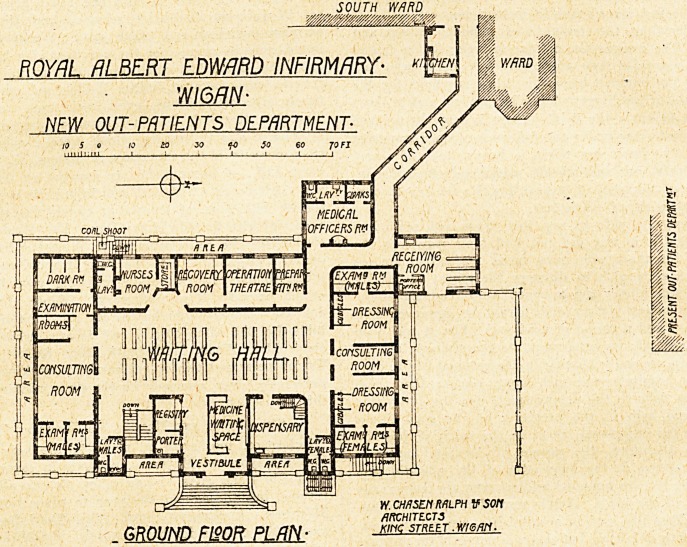


**Figure f2:**